# EEG spectral coherence data distinguish chronic fatigue syndrome patients from healthy controls and depressed patients-A case control study

**DOI:** 10.1186/1471-2377-11-82

**Published:** 2011-07-01

**Authors:** Frank H Duffy, Gloria B McAnulty, Michelle C McCreary, George J Cuchural, Anthony L Komaroff

**Affiliations:** 1 Department of Neurology, Children's Hospital Boston and Harvard Medical School, 300 Longwood Ave, Boston, Massachusetts 02115, USA; 2 Department of Psychiatry, Children's Hospital Boston and Harvard Medical School, 300 Longwood Ave, Boston, Massachusetts 02115, USA; 3 Department of Medicine, Brigham and Women's Hospital and Harvard Medical School, 75 Francis St, Boston, Massachusetts 02115, USA; 4 Department of Medicine, Tufts Medical Center, 800 Washington Street, Boston, Massachusetts 02111, USA

## Abstract

**Background:**

Previous studies suggest central nervous system involvement in chronic fatigue syndrome (CFS), yet there are no established diagnostic criteria. CFS may be difficult to differentiate from clinical depression. The study's objective was to determine if spectral coherence, a computational derivative of spectral analysis of the electroencephalogram (EEG), could distinguish patients with CFS from healthy control subjects and not erroneously classify depressed patients as having CFS.

**Methods:**

This is a study, conducted in an academic medical center electroencephalography laboratory, of 632 subjects: 390 healthy normal controls, 70 patients with carefully defined CFS, 24 with major depression, and 148 with general fatigue. Aside from fatigue, all patients were medically healthy by history and examination. EEGs were obtained and spectral coherences calculated after extensive artifact removal. Principal Components Analysis identified coherence factors and corresponding factor loading patterns. Discriminant analysis determined whether spectral coherence factors could reliably discriminate CFS patients from healthy control subjects without misclassifying depression as CFS.

**Results:**

Analysis of EEG coherence data from a large sample (n = 632) of patients and healthy controls identified 40 factors explaining 55.6% total variance. Factors showed highly significant group differentiation (p < .0004) identifying 89.5% of unmedicated female CFS patients and 92.4% of healthy female controls. Recursive jackknifing showed predictions were stable. A conservative 10-factor discriminant function model was subsequently applied, and also showed highly significant group discrimination (p < .001), accurately classifying 88.9% unmedicated males with CFS, and 82.4% unmedicated male healthy controls. No patient with depression was classified as having CFS. The model was less accurate (73.9%) in identifying CFS patients taking psychoactive medications. Factors involving the temporal lobes were of primary importance.

**Conclusions:**

EEG spectral coherence analysis identified unmedicated patients with CFS and healthy control subjects without misclassifying depressed patients as CFS, providing evidence that CFS patients demonstrate brain physiology that is not observed in healthy normals or patients with major depression. Studies of new CFS patients and comparison groups are required to determine the possible clinical utility of this test. The results concur with other studies finding neurological abnormalities in CFS, and implicate temporal lobe involvement in CFS pathophysiology.

## Background

Fatigue is one of the most common presenting complaints, accounting for a 10-25% prevalence of patients presenting to primary care physicians (PCP) [1]. The extensive differential diagnosis of fatigue encompasses a wide spectrum of illnesses including, but not limited to endocrine disorders, infections, cancer, medication side effects, sleep disorders, seizures, autoimmune diseases, obesity, drug abuse, malingering, and depression [2]. Fortunately, most of these illnesses have characteristic clinical presentations often with confirmatory laboratory tests.

Yet there remain significantly fatigued patients where no underlying diagnosis can be securely established. In the past, such patients were often dismissed as having some form of uncertain psychiatric disorder-typically depression with symptoms of somatization. However, within this 'unclassifiable' but severely fatigued patient population a subset stood out with normal pre-morbid personalities and whose pre-morbid lives were successful and fulfilling. These patients, however, had suddenly become unusually fatigued after an undetermined illness and for whom the subsequent disabling weakness and fatigue endured for more than six months (often years) beyond the resolution of the initial illness. Some, but not all, patients would report intermittent lymphadenopathy and/or low grade fever often with corresponding worsening of their fatigue. Yet, no clear etiology could be found. The term Chronic Fatigue Syndrome (CFS) came to be applied to this group where a suspicion of organic etiology persisted but could not be confirmed [2,3].

Since common psychiatric disorders, particularly depression, often cause fatigue and since psychiatric diagnoses may be difficult to objectively and reliably confirm, many continued to reasonably wonder about the role of an as of yet identified form of depression as the cause of CFS. However, it was found that many patients with CFS suffer from co-existing psychiatric disorders only *after *becoming ill with CFS. Moreover, in 30-50% of patients no co-existing psychiatric disorders [4,5] can be demonstrated. In addition, a carefully controlled trial of fluoxetine in patients with CFS failed to improve fatigue, even in those patients with a concomitant major depression [6].

To better identify this perplexing patient population, the U.S. Centers for Disease Control (CDC) convened a group of experts to establish a set of strict diagnostic criteria for CFS. The resultant criteria have become known as the CDC or Fukuda criteria [3]. These criteria, available as a multi-page evaluation form, serve investigators and clinicians studying CFS to assure that their patient populations are well identified and comparable across studies. CFS is, therefore, not a synonym for prolonged, disabling fatigue although the distinction may be difficult upon initial evaluation. In this paper we use the term CFS to mean CDC-defined CFS.

CFS-which constitutes 0.5-2.5% of primary care referrals and 10-15% of tertiary care referrals for fatigue [1] -remains without confirmatory laboratory tests and can be difficult to distinguish from depression. Between 1 and 8 in 1000 U.S. adults meet the CDC criteria [7]. The CDC estimates that cost to the U.S. economy from lost productivity alone (not including medical care costs) is $9 billion annually [8].

There exists published evidence that CFS may have its underpinnings in organic disease especially within the central nervous system (CNS), although not all studies have found such abnormalities. Studies of the CNS in CFS have included psychometric assessment of cognition [9,10], magnetic resonance imaging [11-13], functional MRI [14,15], *in vivo *MR spectroscopy [16,17], single-photon emission computed tomography [18], positron emission tomography [19], neuroendocrine studies of hypothalamic function [20-22], and studies of the autonomic nervous system [23-25].

A link with infection and CFS also has been reported following infection with Epstein-Barr virus, Ross River virus, *Coxiella burnetii *[26], *Borrelia burgdorferi *[27], parvovirus B19 [28], human herpesvirus-6 [29], and enteroviruses [30]. Novel retroviruses may also be involved [31,32] but that possibility has been challenged [33]. All these infectious agents have the potential to be CNS pathogens. The evidence of neurologic involvement in CFS, and the possible role of infectious agents in triggering and perpetuating CFS, is summarized in a recent review [34].

Symptoms suggesting the possibility of subtle encephalitis in CFS, along with the documented association of CFS with several neurotropic infectious agents, caused us to examine the role of electroencephalographic (EEG) studies in this illness. However, simple visual inspection of EEG has rarely provided valuable information in CFS, aside from allowing exclusion of epilepsy and classic encephalopathy. A study utilizing EEG Spectral Analysis [35] reported no significant differences of spectral power in any EEG frequency bands during sleep between subjects with CFS and their non-fatigued co-twins. Only studies requiring stressful conditions such as repetitive muscular exercise [36] and sleep deprivation [37] have documented EEG spectral difference in CFS.

Accordingly, we undertook an exploration of spectral coherence, a more complex computational derivative of EEG spectral data, which estimates connectivity between brain regions [38-40]. We hypothesized that results would, first, serve to confirm a consistent pattern of brain difference in CFS and, second, provide estimates of the potential for an EEG based diagnostic test for CFS.

## Methods

### Study Population

A total sample of 632 subjects was selected from an existing EEG database of patients referred to and studied at the Developmental Neurophysiology Laboratory, Children's Hospital Boston. Subject groupings, total subjects per group, mean age plus standard deviation per group, and medication status at time of study are shown in Table 1.

**Table 1:** Patient Subgroups

Unmedicated (Total n = 531)		
**Category**	**Total**	**Mean Age (SD)**

Control Females	197	46.5 (18.6)
Control Males	193	44.3 (18.2)
Chronic Fatigue Syndrome Females	38	42.2 (10.6)
Chronic Fatigue Syndrome Males	9	38.6 (11.4)
Depressed Females	10	45.8 (17.6)
Depressed Males	7	47.2 (11.0)
Fatigued Females	60	41.8 (9.3)
Fatigued Males	17	39.7 (8.6)

Medicated (Total n = 101)		

**Category**	**Total**	**Mean Age (SD)**

Chronic Fatigue Syndrome Females	18	43.3 (13.6)
Chronic Fatigue Syndrome Males	5	32.3 (12.4)
Depressed Females	4	37.5 (24.3)
Depressed Males	3	33.9 (5.4)
Fatigued Females	63	41.6 (11.6)
Fatigued Males	8	32.3 (16.2)

Subject Cohort: numbers per group, mean ages (SD = standard deviation).

Controls = Unmedicated, normal control subjects, healthy by physician evaluation,

CFS = Patients meeting CDC criteria for chronic fatigue syndrome, otherwise healthy

Fatigue = Referred patients with non-specific fatigue, CFS status unknown, otherwise healthy

Depression = Physician referred DSM-IV criteria for depression, otherwise healthy.

#### Healthy Controls

A sample of 390 healthy control subjects, all of whom had participated in a large study of normative aging [41,42], served as a control group. All subjects were of normal intelligence, medication free, and screened to exclude past or current medical, neurologic or psychiatric illness. No subject in this group had EEG findings to suggest an underlying seizure disorder or encephalopathic process. The healthy control subjects were divided into two sub-groups, females (n = 197) and males (n = 193).

#### CFS Patients

Seventy patients, all of whom were referred for complaint of disabling fatigue, met the CDC criteria for CFS (the CFS group) [3]. All patients included in this group completed a standardized questionnaire and underwent physical examinations and a battery of laboratory tests to rule out other fatiguing illnesses, and all were classified as having CFS according to an algorithm based on these clinical and laboratory data. EEGs were obtained on patients who reported episodes of impaired cognition (characteristic of the vast majority of patients seen in this practice) and who agreed to undergo the procedure (most of those to whom the procedure was offered). None of the included CFS patients demonstrated clinical or EEG evidence of a seizure disorder. In order to assess the possible effect of psychoactive medications and gender on the EEG results, the subjects were divided into four sub-groups: unmedicated females (n = 38), unmedicated males (n = 9), medicated females (n = 18), and medicated males (n = 5). The CFS group was included to determine and evaluate differences between healthy control subjects and healthy CFS patients.

#### Depression Comparison Group

Twenty-four otherwise medically healthy patients met the DSM-IV criteria for major depression, diagnosed [43] by their referring psychiatrist, blinded to the goals of this study. Disabling fatigue is a characteristic of major depression [44]. All members of this population had been referred for EEG to rule out evidence for seizures and/or encephalopathy and no included patient had EEG evidence to support either diagnosis. These patients were similarly divided into four sub-groups by gender and medication: unmedicated females (n = 10), unmedicated males (n = 7), medicated females (n = 4), and medicated males (n = 3). This group of patients with major depression was included to determine whether a discriminant function developed to distinguish CFS from controls might incorrectly classify depressed patients as having CFS.

#### Patients with Unspecified Fatigue

One-hundred and forty-eight subjects carried the primary complaint of prolonged fatigue of undetermined origin. They were all referred for EEG to rule out an underlying seizure disorder or encephalopathic process. We have no further information beyond the referring physician's written diagnosis. Medication and health status was also determined by routine questionnaire at time of EEG study. Patients who indicated underlying medical disease, who did not confirm fatigue as a primary complaint, and/or with subsequent EEGs showing clear evidence of epilepsy or encephalopathy were excluded. The referring physicians had not rigorously evaluated these subjects by the CDC criteria, and thus it cannot be determined how many had the diagnosis of CFS. This population is most likely comprised of patients with CFS, depression, sleep disorders, and/or other undiagnosed illnesses. This group was similarly divided into unmedicated females (n = 60), unmedicated males (n = 17), medicated females (n = 63), and medicated males (n = 8). This group of patients with unspecified fatigue was included solely to assure adequacy of population variance in the large group of subjects used to develop coherence factors by principal components analysis (PCA) [45].

#### Informed Consent

All participants gave their informed consent in accordance with the protocols approved by the Institutional Review Boards for research with human subjects of the respective referring hospitals, the Brigham and Women's Hospital (BWH), the Massachusetts General Hospital (MGH), and the Children's Hospital Boston (CHB). All subjects were participants in one or more of the following four CHB protocols where EEGs were performed: Computerized Brain Wave Testing; Age Related Changes of Cognition in Health and Disease; Neurophysiology of CFS; QEEG Changes in Patients with CFS.

### Measurements and Data Analyses

#### Methodological issues and solutions

Critiques of neurophysiological investigations typically focus on three potential, methodological sources of error: First, failure to stabilize subject state (e.g., waking, drowsy). Second, failure to remove or otherwise manage classic forms of EEG artifact (e.g., eye movement, eye blink, muscle) with failure to recognize that EEGs appearing clean by visual inspection may yet contain significant artifact [46]. Third, capitalization upon chance-applying statistical tests to too many variables and incorrectly reporting those that appear significant by chance as supporting the experimental hypothesis [45,47-49]. We designed our methods to specifically address these key issues.

#### EEG data collection: Artifact and state management at time of data collection

**Figure 1:**
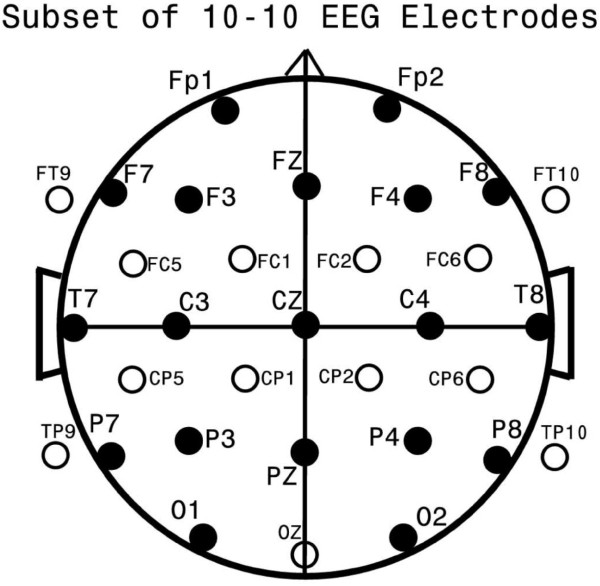
**Standard EEG Electrode Names and Positions**. Head in vertex view, nose above, left ear to left. EEG electrodes: Z: Midline: FZ: Midline Frontal; CZ: Midline Central; PZ: Midline Parietal; OZ: Midline Occipital. Even numbers, right hemisphere locations; odd numbers, left hemisphere locations: Fp: Frontopolar; F: Frontal; C: Central; T: Temporal; P: Parietal; O: Occipital. The standard 19, 10-20 electrodes are shown as black circles. An additional subset of 17, 10-10 electrodes are shown as open circles.

EEG data from all 632 subjects were obtained from 32 gold-cup scalp electrodes affixed by collodion after careful measurement by a registered EEG technologist. Electrode locations, shown in Figure 1, formed a subset of the standard 10-10 EEG electrode locations [50]. EEG data were gathered in the awake, alert, eyes closed state by an EEG technologist, naïve to study goals but specifically trained in the following protocol. Subjects were periodically aroused and given brief breaks either every 1-2 minutes or whenever drowsiness was evident in the EEG-whichever came first. Subjects were then instructed to open their eyes, blink frequently, and move to a comfortable position. Data collection subsequently resumed in the eyes closed state. Data were sampled at 256 Hz after filtering from 1-100 Hz using Grass™ EEG amplifiers, and digitally recorded for subsequent quantitative analyses. All amplifiers were individually calibrated prior to each study. At the end of the data collection, digitized EEG data were visually inspected by the EEG technologist and those EEG epochs during breaks or showing movement artifact, electrode artifact, eye blink storms, drowsiness, and/or bursts of muscle activity were visually identified and eliminated. In our experience expert visual identification of drowsiness has been found equal or superior to detection by automated detection algorithms [51]. EEGs were marked so that all channels during an artifact epoch would be excluded from subsequent analyses. After visual inspection, data were low pass filtered below 50 Hz with an additional 60 Hz mains rejection notch filter. Residual eye blink and eye movement artifacts, which may be surprisingly prominent even during the eyes closed state, were removed using the source component technique [52,53] implemented in the BESA3.5™ software package. Visually, these combined techniques resulted in EEG data that appeared largely artifact free, with rare exceptions of low level temporal muscle artifact and residual frontal and anterior temporal slow eye movement, which remain capable of contaminating subsequent analyses. The final reduction of such residual contamination is discussed below.

#### Calculation of Spectral Coherence Variables

Approximately 15 minutes of EEG collected and processed as noted above were transformed to Current Source Density measures (BESA software), a reference-free condition sensitive to underlying cortex and relatively insensitive to deep/remote EEG sources [54,55]. Spectral coherence measures were derived from the 1-32 Hz range, in 16 two Hz wide spectral bands, resulting in 7936 unique coherence variables. (NB: The 32 by 32 electrode matrix gives 1024 possible coherence values but the matrix diagonal has a value of 1-each electrode to itself-and half of the 992 remaining values duplicate the other half, leaving 496 unique coherences per spectral band. Multiplication by the 16 spectral bands results in 7936 unique spectral coherence values per subject). Coherence data calculation was performed as outlined by Saltzberg [38] using a Nicolet™ software package.

#### Further artifact reduction by multivariate regression

Unfortunately, artifact cannot be removed from an entire EEG data set by direct elimination of electrodes and/or frequencies where a particular artifact may appear most easily seen. For example, although eye blink typically dominates low spectral frequencies in prefrontal regions, its non-sinusoidal waveform will generate harmonics at higher frequencies that will overlap with higher frequency spectral signals that are non-artifactual (brain generated). Furthermore, the spatial field of eye blink can also be expected to involve contamination of more distant electrodes. A similar argument applies to temporal-frontal muscle artifact.

A good approach to further reduce residual artifactual contamination of coherence data involves multivariate regression. Semliltsch has demonstrated [56] that by identifying a signal proportional to a known source of artifact, this signal's contribution to scalp recorded data may be effectively removed by statistical regression procedures. Residual vertical eye movement and blink produce slow EEG delta spectral signals in the frontopolar channels FP1 and FP2 which may be estimated by the average of the 0.5 and 1.0 Hz spectral components from these channels after EEG spectral analysis by Fast Fourier Transform (FFT) [57]. Similarly, horizontal eye movement may be estimated by the average of the 0.5-1.0 Hz spectral components from anterior temporal electrodes F7 and F8. Little meaningful information of brain origin is typically found at this slow frequency in the indicated channels in the absence of extreme pathology (e.g., brain tumors, trauma, and abscess). *Muscle activity*, in contrast, tends to peak at frequencies above those of current interest. Accordingly, 30-32 Hz FFT components were considered to be largely representative of muscle contamination, especially as recorded from the separate averages of prefrontal (FP1, FP2), anterior temporal (F7, F8), mid-temporal (T7, T8), and posterior temporal (P7, P8) electrodes. These electrodes are the most often contaminated by muscle as they are physically closest to the source of the artifact (frontal and temporal muscles). The six artifact measures, two very slow delta and four high frequency beta, were submitted as independent variables to a multiple regression analysis (BMDP2007™-6R) [58] used to individually predict each of the coherence variables (see below) treated as dependent variables. The residuals of this process constitute coherence data that definitionally cannot be predicted by the artifact measures. By adding the residual data from each subject to the original neurophysiologic mean data, artifact free coherence measures were generated which are used for all subsequent analyses.

#### Variable number reduction; creation of coherence factors

Data for all electrodes and for all EEG frequencies produce a large variable number-7936 for our study. To facilitate subsequent statistical analyses, we undertook Principal Components Analysis (PCA) as an objective technique to meaningfully reduce variable number [45]. Our coherence data were first normalized (centered and shifted to have unit variance) so that eventual factors reflect deviations from the average response. To avoid loss of sensitivity by *a priori *data limitation, an 'unrestricted' form of PCA was applied [59] allowing all coherence variables per subject to enter analysis. By employment of an algorithm based upon singular value decomposition (SVD) [59], a data set of uncorrelated (orthogonal) principal components or factors [45,59,60] was developed in which the identification of a small number of factors (following Varimax rotation [61]) describe an acceptably large amount of variance [62]. Varimax rotation enhances factor contrast yielding higher loadings for fewer factors whilst retaining factor orthogonality and has become "...the most widely accepted and employed standard for orthogonal rotation of factors..." (p.145) [63]. Although not the only PCA method applicable to large, asymmetrical matrices (7936 variables by 632 cases as in the current study), SVD (which can be used to solve undetermined and over determined systems of linear equations [57]) is among the most efficient in our experience [59]. This approach to variable number reduction has been successfully used in a prior study of EEG spectral coherence in infants [59].

#### Discriminating groups of subjects by use of EEG spectral coherence variables

Two-group discriminant function analysis (DFA) [63-66] produces a new canonical variable, the discriminant function, which maximally separates the groups and is based on a weighted combination of the entered variables. DFA defines the significance of the group separation, summarizes the classification of each subject, and provides approaches to the prospective classification of subjects not involved in discriminant rule generation by means of the Jackknifing technique [67,68] or by classification of entirely new populations. The BMDP2007™ statistical package [58] was employed for DFA (program 7M). Jackknifing is a technique often used in DFA to estimate prospective classification success [67,68]. In Jackknifing-for two groups DFA as undertaken in this paper-the discriminant function is formed on all subjects but one. The left out subject is subsequently classified. This initial left out subject is then folded back into the group (hence "jackknifing"), another subject is left out, the DFA preformed again, and the newly left out subject classified. This process is repeated until each individual subject has been left out and classified. The measure of classification success is based upon a tally of the correct classifications of the left out subjects. This is frequently referred to as the leaving-one-out process. Alternatively, more than a single subject may be left out for each iteration which may be referred to as the leaving-many-out process. In our experience a more reliable estimate of prospective classification success results from a "leaving 20% out" test. For that reason, we used a random number generator within BMDP-4M (stepwise discriminant analysis) that permits random assignment of each subject to a training set (80% of the subjects, used to create the discriminant) and a test set (20% of the subjects, used to estimate prospective classification). (NB: The algorithm used by BMDP does not always provide a precise 80%/20% split and the ratio of control to experimental subjects within each selected sub-group reflect random chance.) We performed this exercise ten times.

#### Factor description; relating PCA outcome factors to input coherence variables

**Figure 2:**
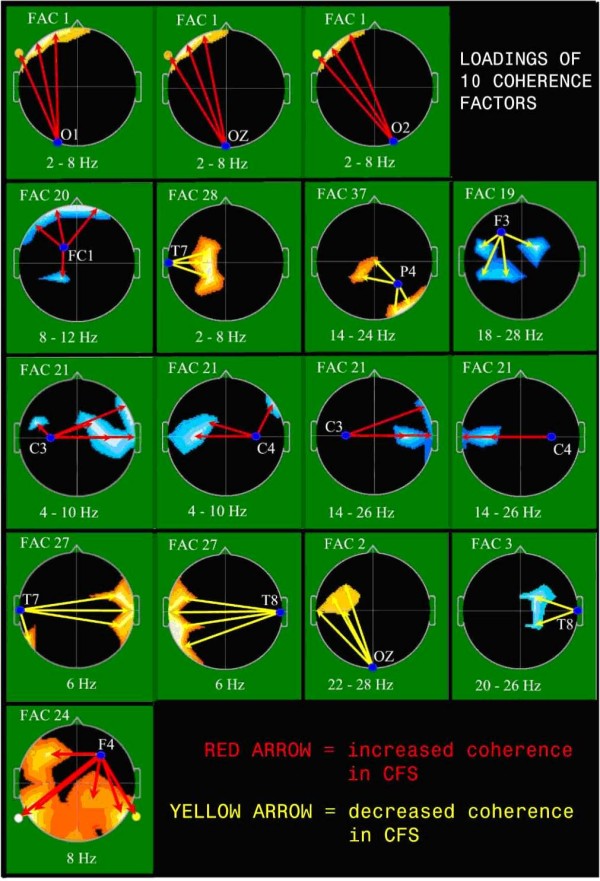
**Graphic Representation of 10 Coherence Factor Loadings**. EEG coherence factor loadings. Heads in top view, scalp left to image left; index electrode within heads and frequency range in Hz below. Region-Colors: Location, magnitude, and sign (red = positive; blue = negative) of maximally loading coherence on factor. Arrow-Colors: Direction of association indicated by arrow (red = increased coherence in CFS; yellow = decreased coherence in CFS).

Individual outcome factors are individually formed as linear combinations of all input variables with the weight or loading of each coherence variable upon a particular factor determined by the PCA computation [63]. As is the general case for PCA, the "meanings" of outcome factors may be discerned by inspection of the loadings of the input variables upon each individual factor [45,63]. To facilitate an understanding of outcome factors for this study, where there are large number of input variables, the factor loadings were treated as if they were primary neurophysiologic data and displayed topographically [69,70]. Display of a representative sample of the highest loading values has typically [71] served to facilitate an understanding of individual factor meaning as shown in Figure 2.

## Results

### Identification and Selection of Spectral Coherence Variables

Variance distribution among the resulting coherence factors was favorable: 2014 factors described over 99%, 302 described 90.03%, 37 described 50.32%, 7 described 26.01% and 1 described 8.25% of the total variance. The first 40 factors-accounting for 55.64% of total variance-were chosen for analysis, exceeding Bartlett's recommendation [72] and resulting in a conservative sample size to variable ratio of 235:40 or 6:1 [73] for the initial DFA described below.

### Discriminating Groups Using Spectral Coherence Variables

The primary discriminant analysis was based on the 197 unmedicated female controls and 38 unmedicated female CFS patients. Female subjects were chosen because in most case series and epidemiologic studies of CFS, females outnumber males [7].

When all 40 coherence factors were forced to enter the DFA, there was a highly significant (p < 0.0004) group differentiation by Wilks' Lambda, with Rao's approximation. The unmedicated female CFS patients were identified with 89.5% accuracy and the female controls with comparable 92.4% accuracy. Age did not significantly differ between these two groups. The statistically significant result, with all 40 factors as variables forced to enter, establishes that these two groups differ on the basis of variables generated from EEG based coherence data.

Stepwise DFA was then utilized to identify a factor subset that best described the group difference. Ten factors (Figure 2, Table 2) entered the model resulting in a highly significant discrimination (p < .001) and equivalent classification success rate: unmedicated female controls 89.85%; unmedicated females with CFS 86.8%. Loadings of the 10 best factors (Table 2) determined to be useful in subsequent group discriminations are topographically displayed in Figure 2.

**Table 2:** Coherence Loadings on 10 Best Coherence Factors

Factor	Loading	Range (Hz)	EEG Electrodes Involved
1	+0.91	2-6	OZ ← → FT9, F7, FP1
		2-6	O1 ← → FT9, F7, FP1
		2-6	O2 ← → FT9, F7, FP1
2	+0.82	24-28	OZ ← → T7, FC5, F3, C3
3	-0.80	20-26	T8 ← → FC6, CP6
19	-0.64	18-28	F3 ← → CP5, FC5, FC1, CP2
27	+0.61	6	T7 ← → P7, FC6, T8, CP6
		6	T8 ← → FC5, T7, CP5, P7
21	-0.61	4-10	C3← → FC5, FC2, C4, T8, F8
		4-10	C4 ← → C3, FC5, F8
		14-26	C3 ← → C4, T8, F8
		14-26	C4 ← → C3, T7
24	+0.58	8	F4 ← → F3, P7, TP9, CP2, P8, TP10
28	+0.57	2-8	T7 ← → FC1, C3, CP1
37	+0.55	14-24	P4 ← → CZ, CP1, O2, P8
20	-0.35	8-12	FC1← → CP1, F7, FP1, FP2

See Figure 1 for scalp location of indicated electrodes.

The results of the 10 jackknifing trials are shown in Table 3. The average success for the ten trials is reported for the control (87.14%) and CFS females (86.2%). Each of these ten iterations generates a unique canonical discriminant variable for each test set member on the basis of the corresponding training set data. As a separate measure of classification success a 2-group analysis of variance (ANOVA) is performed for the discriminant variable on test set subjects (BMDP -7D). All of the 10 iterations reached significance, eight at or below the p < 0.0003 level, one at the p < 0.006 level and one at the p < 0.02 level.

**Table 3:** Recursive Jackknifing by Leaving 20% Out: Test Set Classification Accuracy

Trial	Control	% Correct	CFS	% Correct	*df*	*F*	*p*
1	35/41	85.36	8/9	88.89	1,14	38.09	0.0000

2	34/38	89.47	5/5	100.00	1,5	20.42	0.0063

3	32/39	82.05	9/10	90.00	1,19	39.66	0.0000

4	36/41	87.80	8/9	88.89	1,11	41.38	0.0000

5	37/41	90.24	5/6	83.33	1,6	9.17	0.0232

6	35/39	89.74	8/10	80.00	1,14	22.51	0.0003

7	33/43	76.74	8/9	88.89	1,14	29.51	0.0001

8	41/47	87.23	7/9	77.78	1,11	29.89	0.0002

9	40/44	90.90	11/14	78.57	1,28	51.75	0.0000

10	36/39	92.31	6/7	85.71	1,10	43.47	0.0001

*Mean*		*87.14*		*86.21*			

One-Way Analysis of Variance (F); 2-tailed. Results are number and percent correctly classified of Test Set, *df *= degrees of freedom, p = probability value

By both classification success and ANOVA, results were positive for use of spectral coherence data in prospective classification.

### Applying the Discriminant Function to Other Groups

The 10-factor discriminant function derived from the unmedicated female subjects was then tested on the other patient groups. Of note, 8 of the 9 (88.9%) unmedicated CFS males, whose data were not included in formation of the discriminant formation, were correctly classified.

The discriminant function was applied to male and female CFS subjects who were taking psychoactive medications. Although it performed considerably better than chance, the discriminant performed less well than it had with unmedicated subjects: 14/18 (77.8%) of medicated female CFS patients and 3/5 (60%) of medicated male CFS patients were accurately classified.

For patients with unspecified fatigue whether medicated or unmedicated, 46.6% were assigned to the CFS classification. As the true diagnosis of these subjects is not known, accuracy of the classification cannot be inferred.

Finally, when the discriminant function was applied to all four subgroups of the 24 patients with major depression, none of the depressed patients were falsely classified as having CFS.

### Characteristics of Coherence Variable Differences between CFS and Normal Subjects

There was no clear predominant side (right vs. left) or EEG spectral band involved in the 10 factors that were the best discriminators. However, there were clear differences in the brain regions involved in the ten most discriminating coherence factors, as follows: Temporal region (9/10), central (8/10), frontal (5/10), occipital (3/10), and parietal (1/10) region. (Figure 2)

## Discussion

The *first goal *of this study was to explore meaningful reduction, by principal components analysis (PCA), of a large data set of artifact-free EEG spectral coherence data created from an adult population containing healthy controls and patients with CFS, major depression, and unspecified severe fatigue. Coherence is taken to represent the degree of functional connectivity or coupling between two different brain regions at a chosen frequency.

The *second goal *was to explore the utility of the PCA-reduced data set in differentiating CFS patients from normal subjects without falsely classifying depressed patients as having CFS. Many studies have found evidence of nervous system involvement in CFS, but no large, controlled investigations of the value of EEG spectral coherence in patients with CFS had been reported. Spectral coherence has proven useful in conditions where standard EEG is seldom found to be diagnostic [59,71,74,75].

### First goal, creation of artifact free coherence factors by PCA

Utilizing the full subject population (Table 1, n = 632) we were successful in reducing the initial 7936 coherence variables per subject to 40 orthogonal (uncorrelated) factors per subject which described 55.6% of the total, initial variance. In other words, PCA condensed over half the information (variance) contained in the initial 7936 variables into just 40 new variables (outcome factors). One benefit of this almost 7936:40 or 200 fold reduction in data dimensionality over the entire population is a parallel reduction in the likelihood for capitalization on chance of the sort that may occur during subsequent statistical analyses when they involve large numbers of variables [48]. An additional benefit to this 'hands-off' data reduction is that it requires no advance or *a priori *coherence variable selection by the investigators, eliminating any possible variable selection bias. Bartels refers to this as allowing the intrinsic data structure of the population to select variables [45].

In utilizing this PCA based approach, it is important to include all subjects in the initial PCA, even including subjects with related but not completely defined clinical diagnoses-in our case medicated patients and generally fatigued patients with incomplete diagnoses. Among-subject variance within the population is responsible for factor formation. For instance, had factor formation been limited to healthy normal control subjects exclusively, the degree of variance introduced by fatigue, depression and medications would have, therefore, been absent and factors potentially important to group separation might never have been formed.

Finally, the data underwent an initial multiphase artifact control process (see Methods) performed across the entire population. It is highly unlikely that the final, processed coherence data contained significant eye movement or muscle contamination. Indeed prior to PCA, the coherence data were processed so as to be uncorrelated with six classic measures of eye and muscle artifact. Thus it is unlikely that our study findings reflect artifactual group differences.

Finally, subject selection for the primary study groups (healthy controls, CFS, depression) was rigorous and performed by clinical experts in their fields on the basis of standardized, published criteria. This will facilitate replication including sample selection for future studies here and/or elsewhere.

### Second goal, differentiating CFS patients from healthy controls

Our study findings indicate that EEG spectral coherence data, recorded in the waking eyes closed state, differ significantly between healthy control female subjects and otherwise healthy female patients with CDC-defined CFS. Our 40 coherence factors, significantly separated these two index subject groups at p < 0.001. This fundamental finding indicates that CFS patients manifest patterns of functional brain coupling that differ from those of normal controls. Such a difference of CFS brain physiology may help explain known differences in cognition, memory, sleep, and affect that afflict CFS patients (see Background).

We also found that a small subset of as few as 10 coherence factors were able to accurately identify (by stepwise discriminant analysis) these same unmedicated female subjects (CFS 86.8% accuracy, control 89.8% accuracy). When the rules generated by this analysis on unmedicated females were prospectively applied to unmedicated CFS males and healthy control males who were not involved in the discriminant function creation, true prospective classification accuracy remained high (CFS 88.9%, control 82.4%). In addition, when the classification rules were applied to the entire depressed population, none were falsely, prospectively, classified as having CFS.

Jackknifed classification techniques, employed to provide estimates for the prospective success rate for application of the discriminant rules to new sets of unmedicated female subjects (CFS and normal), was successful. By a re-iterative leaving 20% out processes, accuracy for controls was 87.1% and for CFS was 86.2%, (Table 3). Thus the discriminant should prove effective on entirely new samples. However, that hypothesis must be tested on a large, new set of patients with CFS and comparison groups (healthy and with other fatiguing illnesses) to assure the accuracy and utility of EEG spectral coherence as a diagnostic aid.

### Speculations

The less than 100% accuracy of our spectral coherence based classification function could reflect a deficiency in the CDC criteria for CFS, and/or a deficiency in the coherence-based discriminant itself, and/or unexplored physiological variability even within carefully CDC-defined CFS. For example, multiple etiologic agents have been identified as potential triggers of the CFS phenotype [26], each with the potential for a slightly differing impact upon the central nervous system (CNS) and, hence, on EEG spectral coherence. The possibility of sub-grouping [76] CFS on the basis of coherence and other objective CNS measures (e.g., MRI, SPECT/PET, neuropsychology) may be a fruitful area for further exploration. Subgrouping could result in a broader set of objectively derived CNS measures from neurophysiology and other neuroimaging techniques that might eventually become the diagnostic 'gold standard' for CFS.

When applied to patients with CFS who were taking psychoactive medications at the time of testing, the 10-factor model was less accurate (females, 77.8% accuracy; males, 60.0% accuracy). Since psychoactive medications directly affect the brain, the organ being examined by EEG, it is possible that these medications may modify EEG measures such that their accuracy is compromised. Alternatively, these medications may have had a therapeutic clinical effect on brain function (connectivity), thus causing some CFS patients to electrophysiologically resemble normal controls. Supporting this hypothesis is the observation that some patients were tested while on psychoactive medications because they refused to discontinue them being convinced from past experiences that this might worsen their clinical condition. Thus another fruitful area for further exploration is to determine if EEG spectral coherence is a useful index measure in assessing medication treatment response.

Given a lack of detailed clinical information, it is not possible to determine classification accuracy within our Unspecified Fatigue population. When the 10 coherence factor discriminant is applied to this group 46.6% are classified as CFS. This is broadly consistent with the published estimate that the prevalence of true CFS among patients seeking care from tertiary specialists for prolonged fatigue can be as high as 35% [1].

The finding of bilateral temporal lobe involvement in 9 of 10 factors is of potential clinical significance. The 10 coherence factors did not collectively localize to any other single brain region. This greater temporal lobe involvement is consistent with the global memory impairment in CFS reported by Marcel [9] and Daly [77]. It is also interesting that one neurotropic virus associated with CFS, human herpesvirus-6, appears to selectively affect the temporal lobes and has recently been associated with temporal lobe seizure disorders [78-80].

### Future plans

Our immediate plans call for enlarging our population to prospectively test and refine current findings. This will primarily involve recruiting additional patients with depression and non-CFS prolonged fatigue as well as additional patients with CDC-defined CFS-especially males. All patients will have equivalent evaluations: clinical and behavioral as well and neurophysiological. We plan to evaluate a population of CFS patients before and after beginning medications. We also hope to develop specific classification rules to separate four diagnostic groups: CFS, non-CFS prolonged fatigue, depression, and healthy controls. We plan to search for CFS-gender interactions. All this will require substantially larger populations than now available to us. Finally, within the CFS population we will employ cluster analysis, as successfully applied by Montironi and Bartels [76] in another research area, to search for consistent CFS subpopulations.

## Conclusions

EEG-derived spectral coherence factors accurately classify unmedicated subjects with rigorously-defined CFS, and reliably distinguish them from matched healthy control subjects, while at the same time not falsely classifying depressed patients as having CFS. This finding is in accord with other objective evidence that CFS is associated with organic, brain-based pathophysiology [34]. The discriminant function based on the identified coherence factors is less successful in patients on psychoactive medications, which might reflect a palliative effect of the medications. EEG coherence measures, perhaps in combination with other neuroimaging data, may ultimately prove to provide a valuable diagnostic test for CFS as well as an objective means to evaluate potential CFS therapies.

## Competing interests

The authors declare that they have no competing interests.

## Authors' contributions

The authors' contributions included the following: study concept and design, FD, GM and AK; acquisition of patients, AK and GC; acquisition of the patient data, AK, MM, FD, GM and GC; preparation of neurophysiologic data, FD and MM; analysis and interpretation of the data, FD, GM and AK; and statistical analysis; drafting and revision of the manuscript, FD, GM, AK and MM. FD had full access to all the data in the study and takes responsibility for the integrity of the data and the accuracy of the data analysis. All authors read and approved the final manuscript.

## Pre-publication history

The pre-publication history for this paper can be accessed here:

http://www.biomedcentral.com/1471-2377/11/82/prepub
